# Targeting ferroptosis suppresses osteocyte glucolipotoxicity and alleviates diabetic osteoporosis

**DOI:** 10.1038/s41413-022-00198-w

**Published:** 2022-03-09

**Authors:** Yiqi Yang, Yixuan Lin, Minqi Wang, Kai Yuan, Qishan Wang, Pei Mu, Jingke Du, Zhifeng Yu, Shengbing Yang, Kai Huang, Yugang Wang, Hanjun Li, Tingting Tang

**Affiliations:** 1 Shanghai Key Laboratory of Orthopaedic Implants, Department of Orthopaedic Surgery, Shanghai Ninth People’s Hospital, Shanghai Jiao Tong University School of Medicine, Shanghai, China; 2 Department of Bone and Joint Surgery, Renji Hospital, School of Medicine, Shanghai Jiao Tong University, Shanghai, China; 3 Department of Orthopaedics, Shanghai Jiangong Hospital, Shanghai, China; 4 Department of Trauma Surgery, Department of Orthopedics, Renji Hospital, School of Medicine, Shanghai Jiao Tong University, Shanghai, China; 5 Clinical Stem Cell Research Center, Renji Hospital, Shanghai Jiao Tong University School of Medicine, Shanghai, China

**Keywords:** Diabetes complications, Bone

## Abstract

Diabetic osteoporosis (DOP) is the leading complication continuously threatening the bone health of patients with diabetes. A key pathogenic factor in DOP is loss of osteocyte viability. However, the mechanism of osteocyte death remains unclear. Here, we identified ferroptosis, which is iron-dependent programmed cell death, as a critical mechanism of osteocyte death in murine models of DOP. The diabetic microenvironment significantly enhanced osteocyte ferroptosis in vitro, as shown by the substantial lipid peroxidation, iron overload, and aberrant activation of the ferroptosis pathway. RNA sequencing showed that heme oxygenase-1 (HO-1) expression was notably upregulated in ferroptotic osteocytes. Further findings revealed that HO-1 was essential for osteocyte ferroptosis in DOP and that its promoter activity was controlled by the interaction between the upstream NRF2 and c-JUN transcription factors. Targeting ferroptosis or HO-1 efficiently rescued osteocyte death in DOP by disrupting the vicious cycle between lipid peroxidation and HO-1 activation, eventually ameliorating trabecular deterioration. Our study provides insight into DOP pathogenesis, and our results provide a mechanism-based strategy for clinical DOP treatment.

## Introduction

Diabetic osteoporosis (DOP), a leading cause of fragility fractures and trabecular deterioration in individuals with diabetes, is currently considered a long-term musculoskeletal complication of diabetes mellitus (DM).^1^ Annually, more than 9 million osteoporotic fractures occur worldwide, most of which are associated with DOP. This imposes a major threat to human health and the social economy.^2^ Since glucolipid homeostasis is disrupted in DM, the unique diabetic microenvironment is characterized by abnormally increased levels of extracellular metabolites. Over the past decade, with extensive investigation of the diabetic microenvironment and mineral homeostasis, increased cortical porosity, imbalanced bone metabolism, and distorted bone microarchitecture have been identified as the 3 main characteristics of DOP.^3,4^ Although accumulated evidence from both the bench and the bedside has shown the devastating influence of the diabetic microenvironment on bone metabolism, the underlying pathophysiological mechanisms and effective treatments of DOP remain to be investigated.

Osteocytes are the most abundant cells in mineralized bone tissue. These cells communicate with other bone cells, such as osteoblasts and osteoclasts, via the lacunar-canalicular system and various secreted hormones.^5,6^ A previous study showed that osteocyte function was strongly associated with bone health and that osteocyte death could dramatically destroy the subchondral bone microarchitecture in osteoarthritis.^7^ Moreover, numerous recent studies have shown that osteocytes play a vital role in DOP. Eckhardt et al. first reported that DM led to substantial accumulation of injured osteocytes with a proinflammatory signature.^8^ Yang et al. demonstrated that high glucose significantly inhibited the biomechanical responses of osteocytes by downregulating the expression of the gap junction protein connexin 43 (Cx43).^9^ Despite these findings, the specific mechanism of osteocyte injury in the diabetic microenvironment is still unclear.

Ferroptosis is a new form of programmed cell death induced by uncontrolled iron-dependent lipid peroxidation.^10^ Unlike other forms of cell death, ferroptosis has unique biological hallmarks, such as iron accumulation, increased lipid peroxide production, and downregulation of glutathione peroxidase 4 (GPX4) expression.^11,12^ Accordingly, ferrostatin-1 (Fer-1), a small molecule drug, was found to be an effective ferroptosis inhibitor because of its ability to scavenge lipid peroxides.^13^ Emerging studies have indicated that ferroptosis is involved in metabolic disease, cardiomyopathy, neurodegeneration, ischemia-reperfusion injury, and the effects of cancer immunotherapy.^14–16^ Targeting ferroptosis may be an effective strategy to treat related diseases.

Recently accumulating evidence has confirmed the strong link between glucolipid metabolism and ferroptosis.^17^ The progression of DOP is always accompanied by impaired glucolipid homeostasis and elevated plasma glucolipid metabolites; hence, ferroptosis may play a vital role in the pathogenesis of DOP. However, little is known about the relationship between osteocytes and ferroptosis in the diabetic microenvironment.

In this study, a mouse model of DOP was established, and we confirmed the crucial role of ferroptosis in DOP-induced osteocyte death both in vivo and in vitro. Mechanistically, upregulated HO-1 expression led to heme degradation and intracellular iron overload, triggering lipid peroxidation. This process was dependent on the direct binding between nuclear factor-like 2 (NRF2) and c-JUN. Furthermore, targeting ferroptosis significantly rescued osteocyte death and trabecular deterioration. These results provide insight into the underlying mechanism of DOP and suggest a potential therapeutic target for future DOP treatment strategies.

## Results

### The diabetic microenvironment induced osteocyte ferroptosis in vivo

**Fig. 1:**
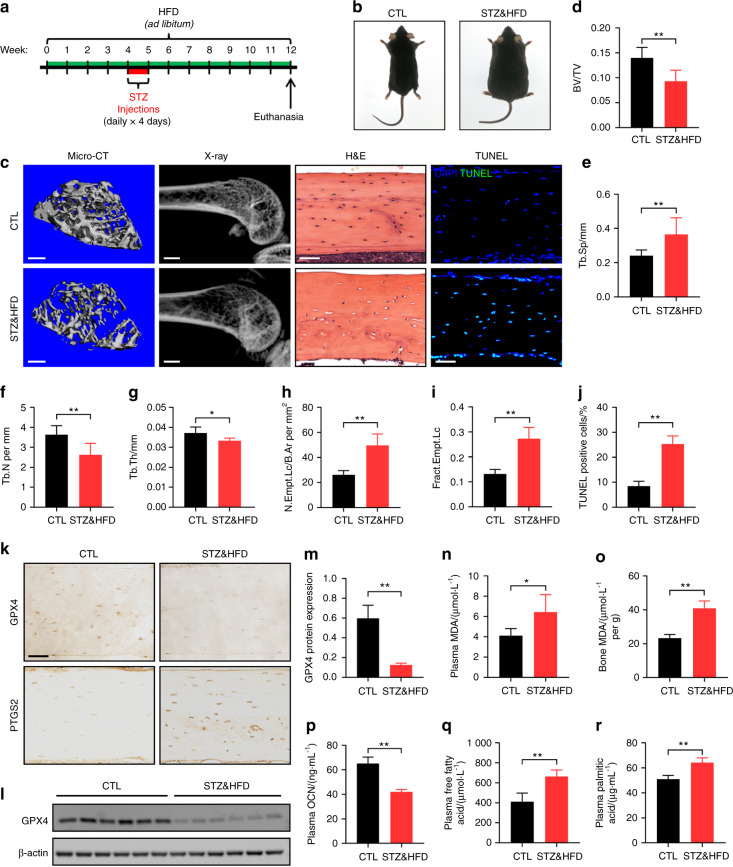
The diabetic microenvironment induced osteocyte ferroptosis in vivo. **a** A schematic illustration of the DOP model established by HFD feeding with injection of low-dose STZ. **b** Gross images of CTL and STZ&HFD mice. **c** Representative micro-CT (scale bar: 300 μm) and X-ray (scale bar: 2 μm) images showing the microarchitecture of the distal femur. Tibial paraffin sections stained with H&E (scale bar: 50 μm) and subjected to TUNEL (scale bar: 50 μm) showed empty lacunae and dead osteocytes. Quantitative analysis of the BV/TV ratio (**d**) trabecular separation (Tb.Sp, **e**), trabecular number (Tb. N, **f**), trabecular thickness (Tb. Th, **g**), number of empty lacunae with respect to bone area (N. Empt. Lc./B. Ar., **h**), fraction of empty lacunae (Frac. Empt. Lc., **i**) and number of TUNEL-positive cells (**j**). **k** Tibial paraffin sections stained for GPX4 and PTGS2 (scale bar: 50 μm). **l** The expression level of GPX4 in tibial bone tissue was determined by WB analysis. **m** Semiquantitative analysis of GPX4 expression based on WB analysis. Concentration of MDA in serum (**n**) and bone tissue (**o**). Serum concentrations of OCN (**p**), FFAs (**q**), and PA (**r**). *P < 0.05; **P < 0.01. Each group contained six mice

To investigate the mechanisms underlying DOP, we first established a mouse model of DOP by administration of a high-fat diet (HFD) and injection of low-dose streptozotocin (STZ) (Fig. 1a). Gross images of the control (CTL) and model (STZ&HFD) mice are presented in Fig. 1b. Plasma glucose and body weight were assessed biweekly, and the fasting insulin concentration was determined on the day of euthanasia. As expected, plasma glucose and body weight were persistently increased in the STZ&HFD mice compared with the CTL mice during the research period (Fig. S1a, b). The elevated fasting insulin indicated possible insulin resistance in the STZ&HFD mice (Fig. S1c). The above results confirmed the successful establishment of the DOP model.

To evaluate the influence of DOP on bone microarchitecture, we performed microcomputed tomography (micro-CT) and X-ray analyses of the distal femur (Fig. 1c). Compared with the CTL mice, the STZ&HFD mice exhibited significant trabecular deterioration and decreased bone mass. As shown in Fig. 1e–g, in the STZ&HFD mice, the bone volume/total volume ratio (BV/TV), trabecular number and trabecular thickness were decreased, while the trabecular separation was increased. However, no significant differences in cortical bone parameters were noted between the CTL and STZ&HFD mice (Fig. S2). To evaluate osteocyte viability in the diabetic microenvironment, we performed hematoxylin-eosin (H&E) staining and terminal deoxynucleotidyl transferase-mediated dUTP nick end labeling (TUNEL) of tibial paraffin sections. In the H&E-stained sections, a large number of empty lacunae were observed in the STZ&HFD group. The fraction of empty lacunae was calculated, as shown in Fig. 1h and i. Moreover, consistent with the H&E staining results, the TUNEL assay also showed that the number of TUNEL-positive cells in the STZ&HFD group was increased approximately 2.5-fold (Fig. 1j).

GPX4 is a lipid hydroperoxidase that decelerates lipid peroxidation, while prostaglandin-endoperoxide synthase 2 (PTGS2) is a proinflammatory mediator that accelerates lipid peroxidation.^18^ Both are closely related to ferroptotic cell death.^19^ Here, immunohistochemical (IHC) analysis of GPX4 and PTGS2 in bone tissues was performed to detect whether ferroptosis occurred in osteocytes (Fig. 1k). Quantitative analysis showed a reduction in GPX4 expression and an obvious increase in PTGS2 expression in the STZ&HFD mice, suggesting that ferroptosis may play an essential role in osteocyte death (Fig. S3). Consistent with the IHC results, western blot (WB) analysis of bone tissue also showed that GPX4 expression was significantly decreased in the STZ&HFD mice (Fig. 1l, m). Then, serum and bone samples were collected from both groups for laboratory testing. Malonaldehyde (MDA) is the final product of lipid peroxidation. As expected, the MDA levels in both serum and bone tissue were significantly elevated in the STZ&HFD group (Fig. 1n, o), indicating that substantial lipid peroxidation occurred in vivo. Osteocalcin (OCN) is the biomarker of active osteogenesis. Compared with that in the CTL group, the serum OCN level was reduced in the STZ&HFD group (Fig. 1o), indicating inhibited bone formation. Interestingly, the levels of free fatty acids (FFAs), especially palmitic acid (PA), were significantly increased in the STZ&HFD mice (Fig. 1p, q). The concentration of PA in the STZ&HFD mice was increased to 65.25 μg·mL^−1^. PA has been reported to be the most abundant saturated FFA and the dominant saturated FFA toxic to skeletal muscle cells.^20^ This finding indicated that high glucose and FFA levels were the main features of the diabetic microenvironment; hence, in the following experiments, we aimed to mimic the diabetic microenvironment by exogenous addition of glucose and PA (HGHF treatment). Taken together, these results indicated that the diabetic microenvironment induced osteocyte ferroptosis in vivo.

### HGHF treatment-induced osteocyte ferroptosis in vitro

**Fig. 2:**
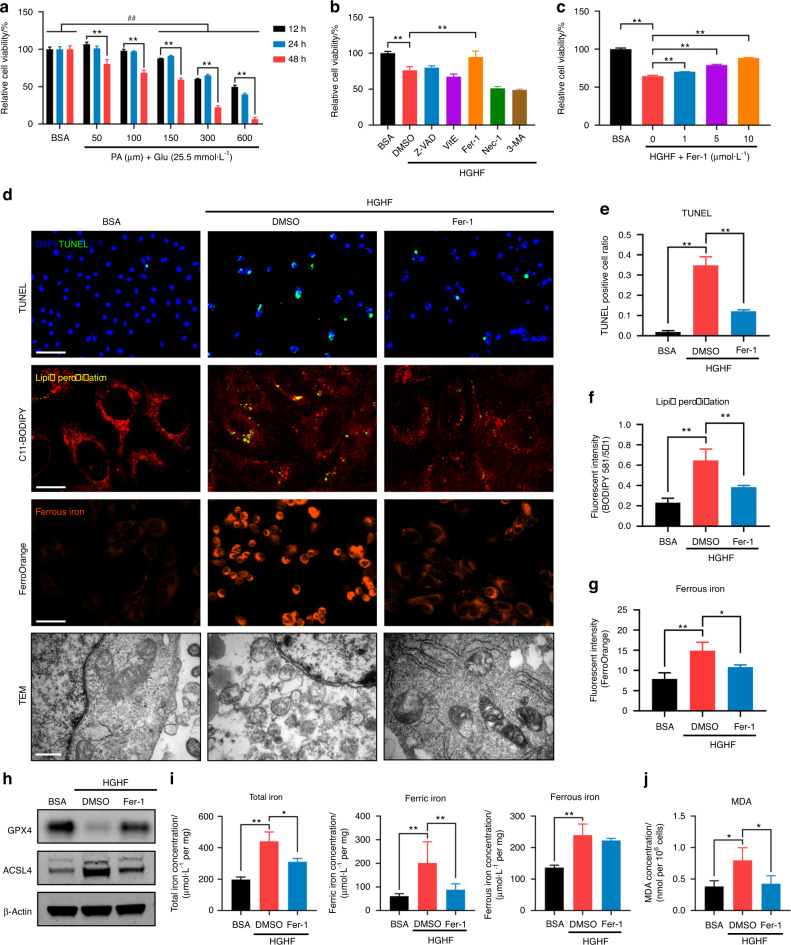
HGHF-induced osteocyte ferroptosis in vitro. **a** Osteocytes were treated with BSA (5.5 mmol·L^−1^ glucose) or various concentrations of PA (25.5 mmol·L^−1^ glucose) for different times and evaluated with a CCK‐8 assay. **b** CCK-8 assay of osteocytes pretreated with DMSO (solvent), Z-VAD-FMK (an apoptosis inhibitor), VitE (a ROS scavenger), Fer-1 (a ferroptosis inhibitor), Nec-1 (a necroptosis inhibitor) or 3-MA (an autophagy inhibitor) and then subjected to HGHF treatment for 24 h. **c** CCK-8 assay of osteocytes pretreated with various concentrations of Fer-1 and then subjected to BSA or HGHF treatment for 24 h. **d** Osteocytes treated with BSA, HGHF or HGHF + Fer-1 for 24 h. Representative images of the TUNEL assay (scale bar: 100 μm), C11-BODIPY staining (scale bar: 25 μm), FerroOrange staining (scale bar: 100 μm), and TEM (scale bar: 0.5 μm) images are presented. **e** Semiquantitative analysis of TUNEL-positive cells. Semiquantitative analysis of the fluorescence intensity of lipid peroxides (**f**) and ferrous iron (**g**). **h** WB analysis of the expression of GPX4 and ACSL4 in osteocytes. **i** Total iron, ferrous iron, and ferric iron levels in osteocytes were quantitatively determined using an iron assay kit. **j** MDA levels in osteocytes were quantitatively determined using an MDA assay kit. “*” indicates comparison between the two indicated groups, and “#” indicates comparison with the BSA group at 24 h. *P < 0.05; “**” and ^##^*P* < 0.01. All data are from *n* = 3 independent experiments

According to previous reports, osteocytes were treated with 25.5 mmol·L^−1^ glucose, which was consistent with the diabetic environment in vivo, while CTL osteocytes were treated with 5.5 mmol·L^−1^ glucose.^21^ Moreover, various PA concentrations were employed separately to determine the optimal inducing concentration (Fig. 2a). We found that HGHF treatment decreased osteocyte viability in a time- and dose-dependent manner. After 24 h of treatment with 300 μmol·L^−1^ PA, osteocyte viability was reduced to 64.88%. In addition, at this concentration, we observed by light microscopy that a great number of osteocytes had collapsed and were floating. When higher PA concentrations were tried, not enough ferroptotic osteocytes could be collected to perform subsequent experiments. Thus, a regimen of PA at 300 μmol·L^−1^ for 24 h was used in this study to facilitate the observation of osteocyte death. Next, to study the relative contributions of different forms of programmed cell death to HGHF-induced osteocyte death, we evaluated the rescue effects of various programmed cell death inhibitors using a CCK-8 assay (Fig. 2b). These inhibitors included Z-VAD-FMK (an apoptosis inhibitor), vitamin E [VitE; a reactive oxygen species (ROS) scavenger], Fer-1 (a ferroptosis inhibitor), necrostatin-1 (Nec-1; a necroptosis inhibitor) and 3-methyladenine (3-MA; an autophagy inhibitor). The results showed that only Fer-1 significantly rescued HGHF-induced osteocyte death, while the other inhibitors showed little effect. Then, we studied the effect of Fer-1 on osteocyte viability at different concentrations. As illustrated in Fig. S4, Fer-1 showed an obvious inhibitory effect at concentrations exceeding 10 μmol·L^−1^. Furthermore, we examined the optimal concentration for rescue of dying osteocytes within the cell-friendly concentration range. The results demonstrated that Fer-1 rescued osteocyte viability in a dose-dependent manner (Fig. 2c); hence, Fer-1 was used at the highest concentration (10 μmol·L^−1^) in the following rescue experiments.

Generally, ferroptosis is accompanied by a series of morphological and biochemical changes, such as mitochondrial deformation, iron overload, and lipid peroxidation. To further confirm the occurrence of ferroptosis, we performed a TUNEL assay, C11-BODIPY staining, FerroOrange staining and transmission electron microscopy (TEM) in HGHF-treated osteocytes with or without Fer-1 rescue (Fig. 2d). Consistent with the CCK-8 assay, the TUNEL assay confirmed that HGHF-induced osteocyte death could be significantly rescued by Fer-1 treatment (Fig. 2e). Intracellular lipid peroxides were detected using the C11-BODIPY fluorescent probe. C11-BODIPY staining showed obvious lipid peroxidation in response to HGHF stimulation, and this effect was rescued by Fer-1 treatment (Fig. 2f). The same trend was also observed in the production of MDA, which is the end product of lipid peroxidation (Fig. 2j).

FerroOrange is an Fe^2+^-specific probe. Its fluorescence intensity increased sharply upon HGHF stimulation, and this change was reversed by Fer-1 treatment (Fig. 2g). Furthermore, the intracellular iron level was quantified using an iron assay kit. The ferrous (Fe^2+^), ferric (Fe^3+^), and total (Fe) iron concentrations increased significantly in response to HGHF and were partially rescued by Fer-1 treatment. Surprisingly, the HGHF + DMSO and HGHF + Fer-1 groups did not show a significant difference in the Fe^2+^ concentration. This result might be due to persistent Fe^2+^ and Fe^3+^ conversion in osteocytes. The morphological features of ferroptosis include a decrease in the mitochondrial size with an increase in the membrane density. By TEM, shrunken mitochondria with ruptured membranes were apparent in the HGHF-treated osteocytes. Fer-1 treatment partially restored the mitochondrial structure. Finally, the results of WB analysis of GPX4 (an anti-ferroptosis protein) and ACSL4 (a pro-ferroptosis protein) are presented in Fig. 2h.

In addition to Fer-1, the iron chelator desferrioxamine (DFO) is a specific ferroptosis inhibitor. Here, we further investigated whether DFO treatment can rescue HGHF-induced osteocyte death. First, we determined the optimal osteocyte rescue concentration of DFO using a CCK-8 assay and found that the rescue effect was best when the DFO concentration was 20 μmol·L^−1^ (Fig. S5a, b). Next, we performed MDA measurement, a TUNEL assay, C11-BODIPY staining, and FerroOrange staining of HGHF-treated osteocytes with and without DFO rescue (Fig. S5c–g). The results demonstrated that DFO inhibited lipid peroxidation and iron accumulation and finally rescued osteocyte death. Taken together, the above results demonstrated that ferroptosis played a crucial role in HGHF-induced osteocyte death in vitro.

### HGHF treatment activated ferroptosis and promoted HO-1 expression in osteocytes

**Fig. 3:**
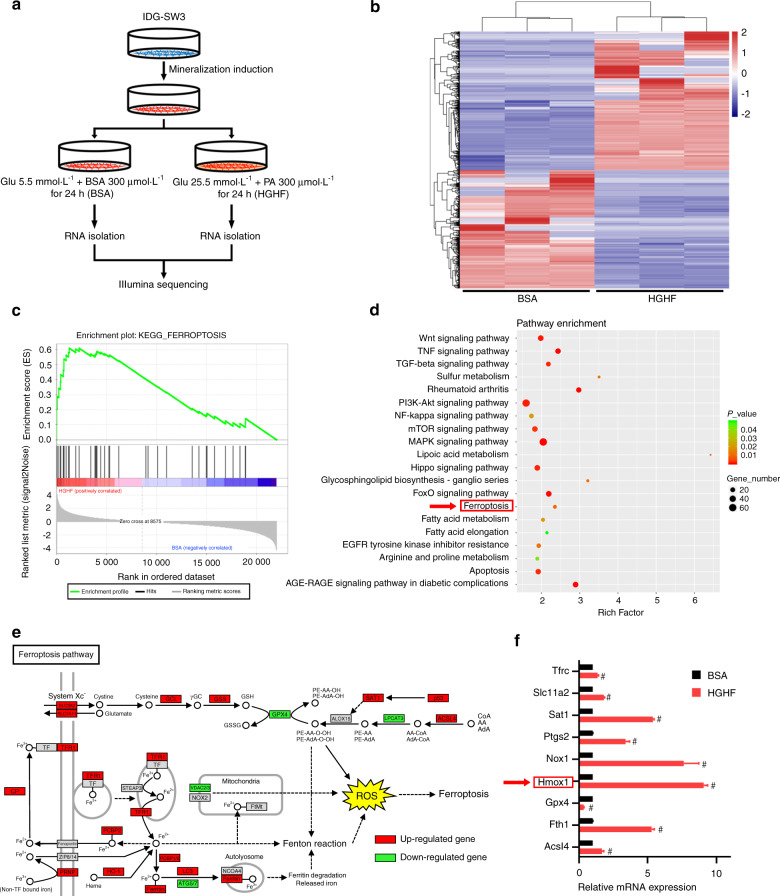
HGHF treatment activated ferroptosis and promoted HO-1 expression in osteocytes. **a** Schematic illustration of the experimental design and procedure for sample preparation for RNA sequencing. **b** Heatmap showing differentially expressed genes between the BSA and HGHF groups of osteocytes. Red: high expression levels. Blue: low expression levels. **c** GSEA enrichment plot for the ferroptosis pathway (normalized enrichment score = 2.11; FDR *q* value < 0.001) based on RNA sequencing. **d** KEGG pathway enrichment analysis of the differentially expressed genes between the BSA and HGHF groups. **e** KEGG database visualization of ferroptosis pathway-associated genes affected by HGHF treatment in osteocytes. Red: high expression levels. Green: low expression levels. **f** The mRNA levels of several ferroptosis-related genes were measured using RT–qPCR in BSA-treated and HGHF-treated osteocytes. The β-actin gene was used as the internal reference gene. ^#^*P* < 0.01. All data are from *n* = 3 independent experiments

To further explore the molecular mechanism underlying HGHF-induced osteocyte ferroptosis, we performed RNA sequencing to determine which genes were differentially expressed between the bovine serum albumin (BSA) and HGHF groups. The experimental design and osteocyte preparation procedure are schematically illustrated in Fig. 3a. Twenty-four hours after HGHF treatment, a total of 2 362 differentially expressed genes (*P* value < 0.05 and fold change >2) were identified, with 1 577 genes showing upregulated expression and 641 genes showing downregulated expression (Fig. 3b, S6). Furthermore, we performed Kyoto Encyclopedia of Genes and Genomes (KEGG) pathway enrichment analysis based on the RNA sequencing data. As expected, the ferroptosis pathway showed a significant change based on the significantly differentially expressed genes (Fig. 3d). A visual representation of the identified ferroptosis-related genes is presented in Fig. 3e. Gene set enrichment analysis (GSEA) also confirmed that the ferroptosis pathway was significantly enriched and activated in the diabetic microenvironment (Fig. 3c). Next, we performed real-time quantitative polymerase chain reaction (RT–qPCR) to verify the RNA sequencing results (Fig. 3f). Among the ferroptosis-related genes, Hmox1 had the most significantly upregulated expression. According to previous studies, Hmox1 plays a crucial role in heme oxidation and iron metabolism, which are closely related to ferroptosis.^22^ Therefore, we speculated that Hmox1 may be essential for HGHF-induced osteocyte ferroptosis.

### HO-1 was essential for HGHF-induced ferroptosis

**Fig. 4:**
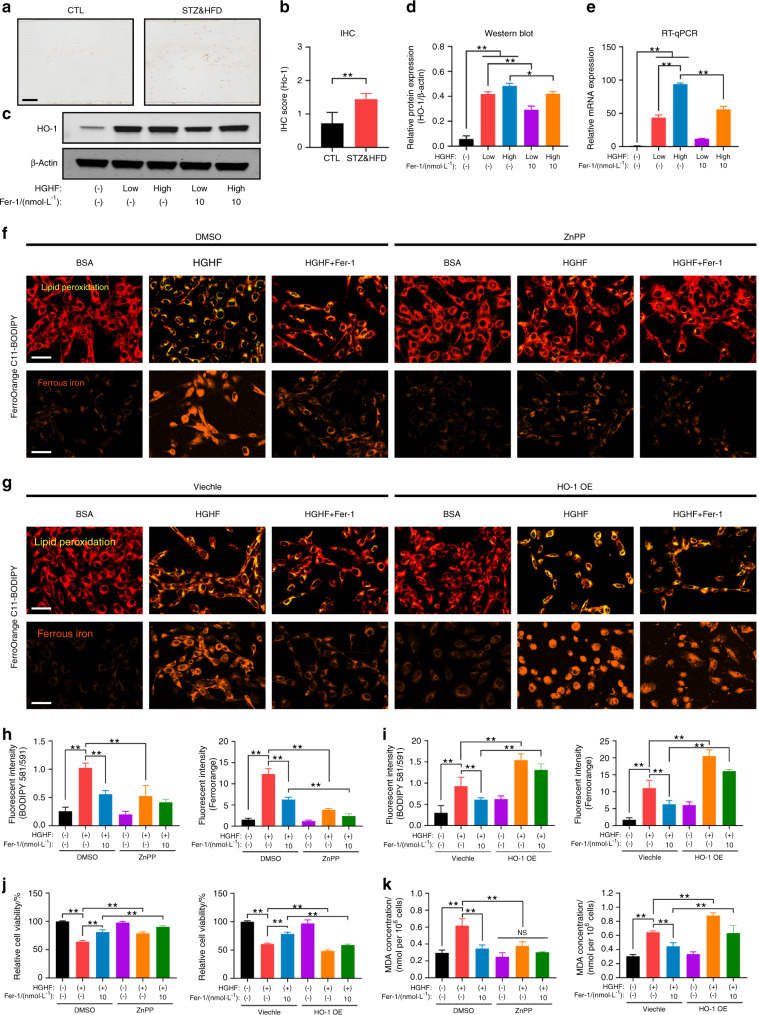
HO-1 was essential for HGHF-induced ferroptosis. **a** Representative images of HO-1-stained bone sections from CTL mice and STZ&HFD mice (scale bar: 50 μm). **b** Quantification of HO-1 expression in vivo. **c** Western blot showing HO-1 expression under various treatments. **d** Quantification of HO-1 expression based on WB analysis. **e** RT–qPCR showing HO-1 expression under various treatments. The β-actin gene was used as the internal reference gene. C11-BODIPY (scale bar: 100 μm) and FerroOrange (scale bar: 100 μm) staining of ZnPP-treated osteocytes (**f**) and HO-1-overexpressing osteocytes (**g**). **h**, **i** Quantification of fluorescence intensity. **j** Osteocyte viability was assessed by a CCK-8 assay. **k** The MDA concentration was assessed using an MDA assay kit. **P < 0.01. “NS” indicates nonsignificant. All data are from *n* = 3 independent experiments

To verify the above hypothesis, we first analyzed HO-1 expression in osteocytes and bone tissues. As shown in Fig. 4a, b, the number of HO-1-positive osteocytes was significantly increased in STZ&HFD mice compared with CTL mice. Consistent with the in vivo results, the in vitro expression of HO-1 in IDG-SW3 cells also increased sharply in response to HGHF treatment in a dose-dependent manner, and this change was partially reversed by Fer-1 (Fig. 4c, d). In addition, the mRNA level of HO-1 showed the same trend as its protein level (Fig. 4e).

To explore the functional role of HO-1 in the pathogenesis of HGHF-induced ferroptosis and DOP, we treated osteocytes with zinc protoporphyrin IX (ZnPP; a competitive Hmox1 inhibitor). The HGHF-induced increases in intracellular lipid peroxidation and iron accumulation were strongly blocked by ZnPP (Fig. 4f). Next, we performed HO-1 protein overexpression (HO-1 OE) in osteocytes using the pcDNA3.1(+) expression plasmid and verified the transfection efficiency by WB analysis (Fig. S7). In contrast to inhibitor treatment, HO-1 OE significantly exacerbated HGHF-induced lipid peroxidation and iron accumulation in osteocytes, even blocking the effect of Fer-1 (Fig. 4g). The quantitative analysis of the fluorescence intensity is presented in Fig. 4h, i. Moreover, increased osteocyte injury with lipid oxidative stress was observed along with the HGHF-induced changes in cell viability and MDA production, and these changes were blocked by ZnPP but exacerbated by HO-1 OE (Fig. 4j, k). Thus, the results of both inhibition and activation of HO-1 suggested its essential role in HGHF-induced osteocyte ferroptosis and DOP.

### HGHF enhanced the NRF2–c-JUN interaction

**Fig. 5:**
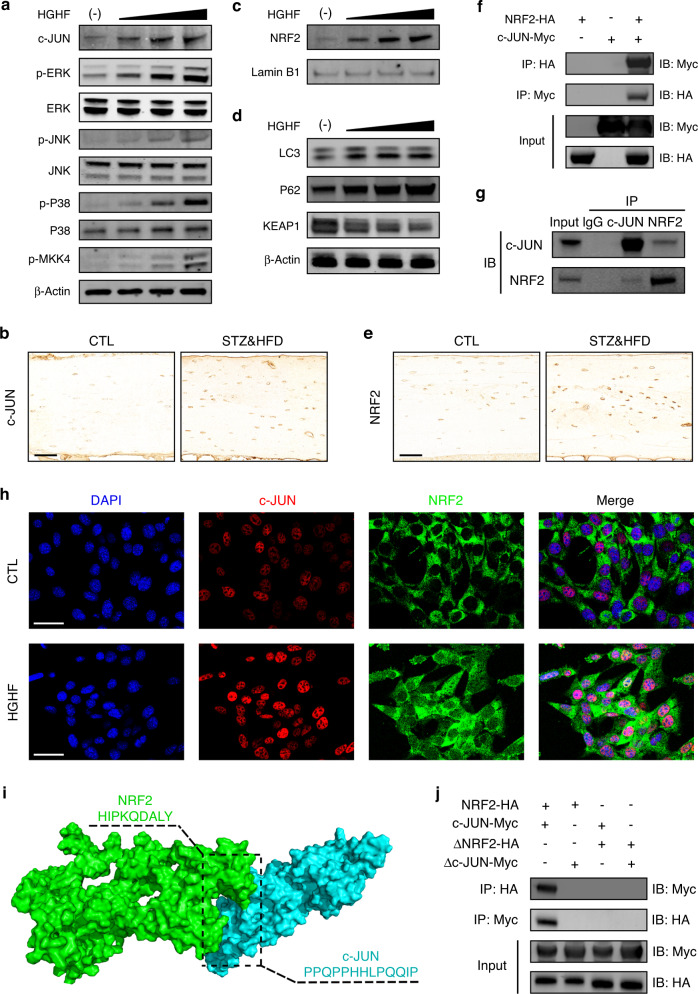
HGHF enhanced the NRF2–c-JUN interaction. **a** Effects of HGHF on the levels of MAPK signaling pathway proteins, including p-MKK4, p‐p38/p38, p‐JNK/JNK, p‐ERK/ERK, and c-JUN. **b** Representative images of bone sections stained with an anti-c-JUN antibody (scale bar: 50 μm). **c** Effects of HGHF on nuclear import of NRF2. **d** Effects of HGHF on NRF2 upstream signaling pathway proteins, including LC3, P62 and KEAP1. β-Actin was used as the internal control for cytoplasmic proteins, and Lamin B1 was used as the internal control for nuclear proteins. **e** Representative images of bone sections stained with an anti-NRF2 antibody (scale bar: 50 μm). **f** Co-IP results for NRF2 and c-JUN in osteocytes transfected with the NRF2-HA and c-JUN-Myc plasmids. **g** Co-IP results for endogenous NRF2 and c-JUN in osteocytes. **h** IF showing that NRF2 colocalized with c-JUN in the nucleus in osteocytes under HGHF treatment (scale bar: 50 μm). **i** 3D binding structure of NRF2 and c-JUN determined via molecular modeling and docking studies. **j** Co-IP results for ΔNRF2 and Δc-JUN in osteocytes transfected with plasmids expressing the NRF2-HA and c-JUN-Myc truncations. All data are from *n* = 3 independent experiments

As mentioned above, we explicitly demonstrated the essential role of HO-1 in osteocyte ferroptosis, but how HO-1 expression is upregulated in response to HGHF treatment is still unclear. Previous studies have reported that HO-1 expression may be modulated by the mitogen-activated protein kinase (MAPK) and NRF2 signaling pathways.^23,24^ Therefore, we speculated that HO-1 expression could be driven by the MAPK and NRF2 signaling pathways in the diabetic microenvironment. To verify this hypothesis, we first analyzed the protein expression of the major components of the three MAPK signaling cascade (ERK, JNK, and p38), an upstream kinase (MKK4) and a major transcription factor (c-JUN) under HGHF treatment. As shown in Fig. 5a, HGHF treatment dose-dependently promoted the phosphorylation of ERK, JNK, p38, and MKK4. More importantly, HGHF also directly upregulated c-JUN protein expression. IHC staining of osteocytes in vivo also showed increased c-JUN expression in the STZ&HFD group (Fig. 5b). NRF2 is a well-known antioxidant-inducible transcription factor. The function of NRF2 is dependent on the degradation of its endogenous inhibitor, KEAP1, and its subsequent translocation into the nucleus.^25^ As shown in Fig. 3d, HGHF treatment dose-dependently downregulated KEAP1 expression, with obvious activation of the autophagy-related proteins LC3 and P62. Furthermore, WB analysis of isolated nuclear proteins showed that HGHF significantly promoted the nuclear translocation of NRF2 (Fig. 5c). Consistent with the finding in vitro, NRF2 expression was also upregulated in STZ&HFD mice (Fig. 5e). The quantitative analysis of c-JUN and NRF2 expression in vivo is presented in Fig. S8. The above results indicated that HGHF treatment activated the MAPK and NRF2 signaling pathways in ferroptotic osteocytes.

Generally, once translocated into the nucleus, activated NRF2 forms heterodimers with specific sMaf proteins, and this dimerization process is necessary for DNA binding. Thus, we speculated that NRF2 can directly bind to c-JUN to form a heterodimer in osteocytes in response to HGHF treatment. To evaluate this ability, we performed coimmunoprecipitation (Co-IP) and immunofluorescence (IF) assays. As shown in Fig. 5f, an interaction between NRF2 and c-JUN was observed in osteocytes transfected with the NRF2-HA and c-JUN-Myc plasmids. Next, an endogenous Co-IP assay again demonstrated the interaction between NRF2 and c-JUN in osteocytes under HGHF treatment (Fig. 5g). Notably, the endogenous direct binding between NRF2 and c-JUN was abrogated in the absence of HGHF treatment, although a slight interaction was still observed in transfected osteocytes (Fig. S9). This finding indicated that the binding of NRF2 and c-JUN was partially dependent on HGHF stimulation. Moreover, IF staining revealed increased colocalization of NRF2 and c-JUN in response to HGHF treatment (Fig. 5h).

Furthermore, to identify the specific binding sites between the two proteins, we constructed a model of the proposed NRF2:c-JUN heterodimer by molecular modeling and docking (Fig. 5i). Based on our docking and protein–protein interface analyses, we found that amino acids 107–115 of NRF2 and amino acids 207–219 of c-JUN might be potential binding domains. To verify the predicted binding sites, we constructed plasmids expressing a truncated NRF2 mutant (ΔNRF2, lacking amino acids 107–115) and a truncated c-JUN mutant (Δc-JUN, lacking amino acids 207–219) for a Co-IP assay. As shown in Fig. 5j, no direct binding was detected in the truncation groups. Taken together, the above results indicated that HGHF enhanced NRF2:c-JUN heterodimer formation in osteocytes, and amino acids 107–115 of NRF2 and 207–219 of c-JUN might be essential for their direct binding.

### HGHF-induced HO-1 expression was dependent on the NRF2–c-JUN interaction

**Fig. 6:**
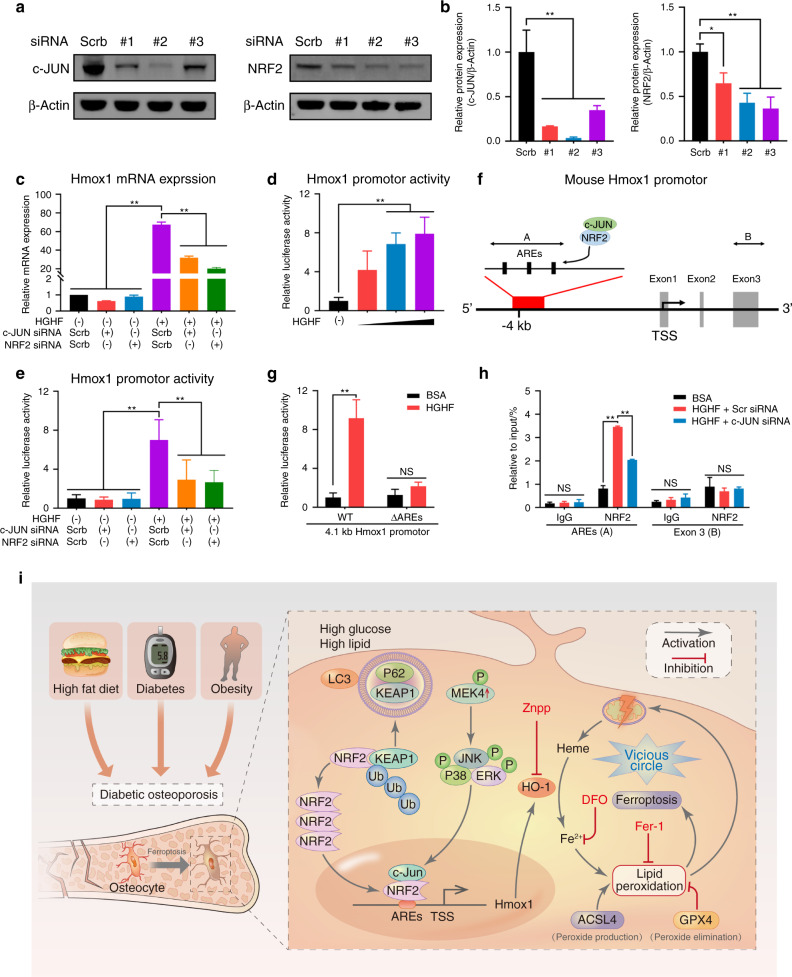
HGHF-induced HO-1 expression was dependent on the NRF2–c-JUN interaction. HGHF-induced HO-1 expression was dependent on the NRF2–c-JUN interaction. **a** WB results for osteocytes transfected with the indicated siRNA or scrambled siRNA. **b** Semiquantitative analysis of WB results. **c** Hmox1 mRNA expression under the indicated treatments was determined using RT–qPCR. **d** Hmox1 promoter activity under treatment with various concentrations of HGHF was assessed using a dual-luciferase reporter assay. **e** Hmox1 promoter activity under the indicated treatments was assessed using a dual-luciferase reporter assay. **f** Schematic representation of the mouse Hmox1 promoter region, including the AREs. **g** Osteocytes were transfected with the −4 100/+50 promoter construct (WT) or a mutant −3 900/+50 construct (with deletion of the AREs, ΔAREs), and Hmox1 promoter activity was assessed using a dual-luciferase reporter assay. **h** Osteocytes were treated as indicated for 24 h, and ChIP assays were performed with an anti-NRF2 antibody or normal IgG to detect AREs. **i** Schematic illustration of the proposed molecular mechanism underlying osteocyte ferroptosis in DOP. HGHF-induced HO-1 expression was dependent on the NRF2–c-JUN interaction. *P < 0.05; **P < 0.01. “NS” indicates nonsignificant. All data are from *n* = 3 independent experiments

To further explore the molecular mechanism underlying the promotion of HO-1 expression by the NRF2:c-JUN heterodimer, we transfected osteocytes with NRF2 or c-JUN siRNA to knock down the expression of these genes (Fig. 6a). As shown in Fig. 6b, the 2nd siRNA sequence targeting c-JUN and the 3rd siRNA sequence targeting NRF2 showed the highest knockdown efficiencies; hence, these sequences were used in the following experiments. Then, Hmox1 mRNA expression was determined. As demonstrated in Fig. 6c, HGHF promoted Hmox1 mRNA expression, as expected, but knockdown of either NRF2 or c-JUN significantly reversed the increase in Hmox1 expression in the diabetic microenvironment.

Furthermore, the effect of the NRF2:c-JUN heterodimer on the transcription of Hmox1 was investigated. To this end, a luciferase reporter plasmid was constructed using a HO-1 promoter fragment (−4 100/+50) containing the E1 enhancer region, which is a major regulator of Hmox1 transcription.^26^ As shown in Fig. 6d, HGHF treatment dose-dependently stimulated Hmox1 promoter-driven luciferase expression in osteocytes. More importantly, knockdown of either NRF2 or c-JUN obviously inhibited HGHF-induced luciferase expression (Fig. 6e). Next, we found that the promoter region of Hmox1 contains three antioxidant response elements (AREs), which have been reported to be crucial transcriptional regulators that bind to NRF2 (Fig. 6f).^27^ To investigate the essential role of these AREs in activating the Hmox1 promoter, we constructed a plasmid expressing a mutant (ΔAREs) in which the three AREs were deleted. As shown in Fig. 6g, HGHF treatment no longer significantly promoted Hmox1 transcriptional activity in ΔAREs cells, confirming that the HGHF-induced increase in Hmox1 transcription was dependent on the functionality of the AREs in the promoter region. Finally, chromatin immunoprecipitation (ChIP) assays were performed using osteocytes. Fragments of the regions containing the AREs were precipitated by the anti-NRF2 antibody, and HGHF treatment significantly promoted the recruitment of NRF2 to the AREs, demonstrating that NRF2 directly binds to this region of chromatin (Fig. 6h). However, this precipitation was partially abrogated by c-JUN deficiency, suggesting that c-JUN is essential for NRF2:c-JUN heterodimer binding to AREs. Taken together, these results demonstrated that HGHF-induced HO-1 expression was dependent on the NRF2–c-JUN interaction (Fig. 6i).

### Targeting the ferroptosis pathway rescued DOP and osteocyte death in diabetic mice

**Fig. 7:**
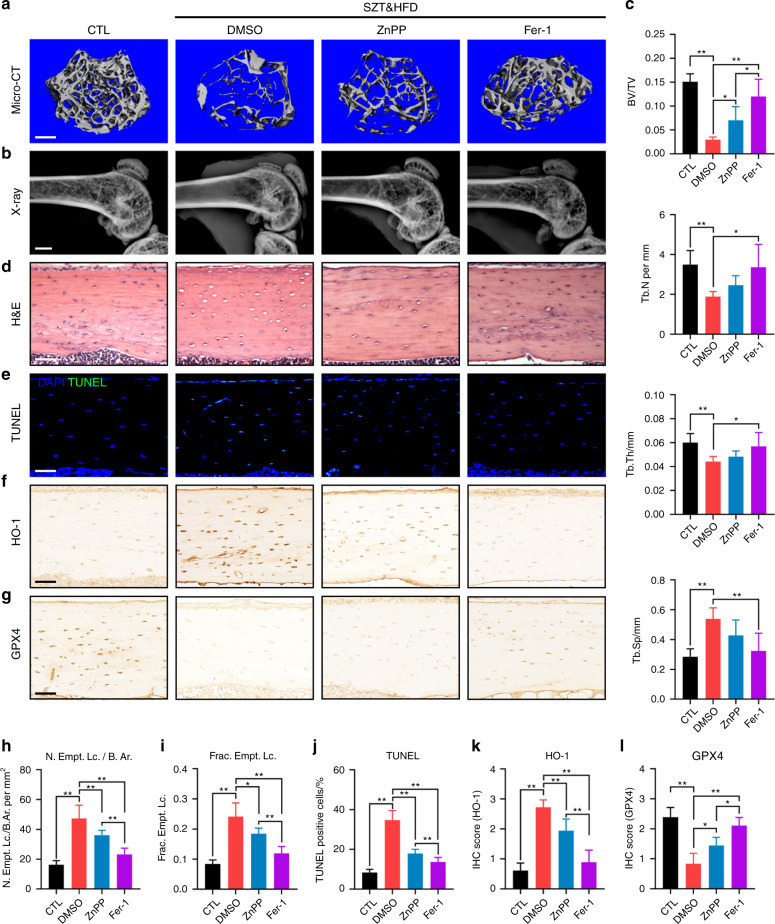
Targeting ferroptosis rescued DOP and osteocyte death in diabetic mice. **a**, **b** Representative micro-CT (scale bar: 300 μm) and X-ray (scale bar: 2 μm) images showing the microarchitecture of the distal femur. **c** Quantitative analysis of the micro-CT parameters. **d** H&E staining of tibial paraffin sections showing empty lacunae (scale bar: 50 μm). **e** TUNEL assay of tibial paraffin sections showing dead osteocytes (scale bar: 50 μm). **f**, **g** Tibial paraffin sections stained for HO-1 and GPX4 (scale bar: 50 μm). **h**, **i** Quantitative analysis of the number of empty lacunae with respect to the bone area (N. Empt. Lc./B. Ar.) and the fraction of empty lacunae (Frac. Empt. Lc.) based on H&E staining. **j** Quantitative analysis of TUNEL-positive cells based on a TUNEL assay. **k**, **l** Quantitative analysis of HO-1 and GPX4 expression in vivo based on IHC staining. *P < 0.05; **P < 0.01. Each group contained six mice

To confirm our findings in animal experiments, we administered ZnPP (an HO-1 inhibitor) and Fer-1 (a ferroptosis inhibitor) intraperitoneally (*i.p*.) to STZ&HFD-induced DOP mice. Since DFO is a small water-soluble molecule whose direct injection leads to its rapid degradation, we did not use DFO in these animal experiments. As shown in Fig. 7a–c, STZ&HFD-induced trabecular deterioration and bone loss were significantly rescued by ZnPP and Fer-1 treatment. Interestingly, Fer-1 treatment consistently had a better therapeutic effect than ZnPP treatment, suggesting that scavenging of intracellular lipid peroxides by Fer-1 may be a better therapeutic strategy for DOP. In addition to trabecular homeostasis, lacunar emptying and osteocyte death were also reversed by ZnPP and Fer-1 treatment in diabetic mice, as shown by H&E staining and a TUNEL assay (Fig. 7d, e). The therapeutic effect of Fer-1 was still quantitatively better than the effect of ZnPP, as expected (Fig. 7h–j). At the molecular level, we detected the expression of HO-1 and GPX4 in tibial paraffin sections from the different groups (Fig. 7f, g). As shown in Fig. 7k, both ZnPP and Fer-1 treatment significantly downregulated HO-1 expression in diabetic mice, which disrupted the vicious cycle of HO-1-mediated ferroptosis. Moreover, the expression of GPX4 in diabetic mice was obviously upregulated by ZnPP and Fer-1 treatment, suggesting that ZnPP and Fer-1 can restore redox homeostasis and enhance ferroptosis resistance in osteocytes. Taken together, these data showed that targeting the ferroptosis pathway was a feasible strategy for rescuing DOP and osteocyte death in diabetic mice.

## Discussion

During the progression of DOP, excessive glucolipid metabolites accumulate in bone tissue, reprogramming bone cell metabolism.^20^ The deleterious effects of the diabetic microenvironment on bone strength and bone mass have recently gained more recognition.^21^ However, the underlying mechanism of osteocyte death in the diabetic microenvironment has not been fully elucidated, and no effective therapeutic strategy is currently available for DOP. Herein, substantial osteocyte death with obvious ferroptotic phenotypes, including lipid peroxide accumulation, morphological changes in mitochondria, iron overload, and ferroptotic gene activation, was observed in DOP. Then, we demonstrated that the inducible heme oxygenase HO-1 played an essential role in osteocyte ferroptosis and that the diabetic microenvironment promoted its transcription by increasing the interaction between NRF2 and c-JUN. Notably, targeting either ferroptosis or HO-1 significantly reversed DOP-related bone loss and osteocyte death, further disrupting the vicious cycle of ferroptosis to restore the intracellular redox balance (Fig. 6i).

The concept of DOP was first proposed in 1984 and is currently considered a prevalent type of secondary osteoporosis.^28^ Interestingly, bone mineral density is significantly decreased in type 1 DOP but not in type 2 DOP. In addition, compared to type 1 DOP, type 2 DOP shows more severe bone microarchitecture deterioration characterized by increased cortical porosity and bone fragility. Therefore, the mechanism underlying type 2 DOP might be more complex and deserves further investigation. In this study, a mouse model of type 2 DOP was established using a HFD and injection of low-dose STZ. Consistent with findings in other DOP models, no cortical parameter changes were observed in our micro-CT analysis.^21,29^ One possible explanation is that 8 weeks of modeling was insufficient to allow cortical bone remodeling. Moreover, a significant reduction in the BV/TV of trabecular bone was found in STZ&HFD mice. This result was consistent with previous findings showing that glucolipotoxicity may first affect the most active region of bone remodeling.^30,31^

Trabecular deterioration in STZ&HFD mice is believed to result from inhibited bone formation and/or excessive bone resorption. Numerous studies have reported that hyperglycemia and hyperlipidemia can directly inhibit osteogenesis in osteocyte precursor cells (osteoblasts), but the detailed pathway by which this process occurs is still unclear.^32^ In contrast to bone formation, opinions vary greatly about the effect of the diabetic microenvironment on bone resorption. Long-term HFD consumption alone has no effect on osteoclastogenesis, but HFD consumption combined with elevated blood glucose can significantly increase osteoclast number and activity.^33,34^ However, it is quite difficult to determine whether this pro-osteoclastogenic effect originates directly from glucolipid metabolites or arises from the secretion of damage-associated molecular patterns by dying osteocytes in the diabetic microenvironment.^35,36^

In addition to trabecular deterioration, osteocyte death was another significant finding in this study. Maintaining osteocyte viability is considered an effective strategy to maintain bone health.^37–39^ In previous studies, caspase-dependent apoptosis and necrosis were regarded as the major forms of osteocyte death. For example, Emerton *et al*. reported that estrogen loss increased osteocyte apoptosis, further decreasing bone volume.^40^ However, in our study, we found that classical cell death inhibitors, such as Z-VAD-FMK and Nec-1, had no effect on HGHF-induced osteocyte death and that only Fer-1 showed an effective rescue effect. Based on these results, we realized that excessive lipid peroxidation could be the major cause of cell injury in the diabetic microenvironment and that ferroptosis might be closely related to the underlying molecular mechanism.

Fer-1, which is a synthetic compound, acts as an efficient hydroperoxyl radical scavenger in the presence of reduced iron, producing an antiferroptotic effect similar to that of GPX4. The scavenging action of Fer-1 occurs in two steps: (i) reduction of alkoxyl radicals by Fer-1 and (ii) reduction of oxidized Fer-1 by ferrous iron.^13^ This explained the decrease in ferrous iron in the Fer-1 rescue group. Notably, Fer-1 works in a paracatalytic manner and is not consumed as it inhibits lipid peroxidation. In contrast to Fer-1, DFO is an iron-chelating agent that inhibits ferroptosis by binding to intracellular free labile iron. Therefore, compared with DFO, Fer-1 displays better rescue effects. Moreover, unlike other cell death inhibitors that target specific proteins in signaling pathways, Fer-1 directly scavenges intracellular lipid peroxides, rescuing osteocytes from the source of injury. Thus, considering the common pathogenesis of metabolic diseases, we speculated that targeting lipid peroxidation with Fer-1 could also provide a feasible therapeutic approach for other diseases.

HO-1 is a cellular inducible oxidative stress regulator that oxidizes heme to yield biliverdin, carbon monoxide, and free ferrous iron.^23^ ZnPP is a specific HO-1 inhibitor and competitively inhibits the binding of heme. Currently, the function of HO-1 in ferroptosis is still under debate. Several studies demonstrated that increased HO-1 expression protected cells from ferroptosis by suppressing oxidative stress.^41^ For example, Adedoyin et al. reported that compared to HO-1^+/+^ renal proximal tubule cells, HO-1^−/−^ cells showed increased erastin-induced cell death.^42^ However, other researchers found that overexpressed HO-1 exacerbated ferroptosis and induced organ failure. Fang et al. reported that suppressing HO-1 expression helped mitigate ferroptosis in cardiomyopathy both in vivo and in vitro.^14^ Tang et al. found that inhibiting HO-1 activity was a robust and efficient method of protecting the retinal pigment epithelium from ferroptosis.^43^ These studies indicated that HO-1 is a double-edged sword, playing various roles in different tissues and different disease models.

Our findings support the idea that HGHF-induced osteocyte ferroptosis is mediated by HO-1, but whether HO-1 activation is only a cause or also a consequence cannot be ascertained. In this study, we found that the connection between HO-1 activation and ferroptosis can cause a vicious cycle of mutual promotion and that both factors are mutually causative. First, activated HO-1 in the diabetic microenvironment catalyzes heme oxidation to release substantial amounts of free labile iron. Then, this iron catalyzes the Fenton reaction, in which accumulated lipid acids react with ROS, forming lipid peroxides and lipid peroxyl radicals. Consequently, the large amount of lipid peroxides formed oxidizes the cell membrane and organelles, releasing more heme and ROS from damaged mitochondria. In this way, a vicious ferroptotic cycle is formed (Fig. 6i). Based on our in vitro and in vivo rescue experiments, we finally confirmed that HO-1 activation and lipid peroxidation are two key steps in the vicious cycle of DOP.

This study indeed has certain limitations. Even though DOP bone specimens are difficult to collect, the use of human tissues and primary cells may further validate our conclusions. Moreover, we confirmed that HO-1 is crucial for osteocyte ferroptosis in the diabetic microenvironment, but the interactions of HO-1 with other classical ferroptosis biomarkers are still unclear. For example, we also noted changes in the expression of GPX4 and ACSL4 in the diabetic microenvironment. In the future, we will seek to unveil the inherent connections among GPX4, ACSL4 and HO-1 in ferroptotic osteocytes.

In conclusion, we illustrated the molecular mechanism by which ferroptosis mediates the pathogenesis of osteocyte death and DOP through aberrant HO-1 activation both in vitro and in vivo. Our findings indicated that the NRF2:c-JUN heterodimer is the upstream activator of HO-1 transcription in the diabetic microenvironment. Moreover, targeting ferroptosis or HO-1 could efficiently rescue osteocyte ferroptosis in DOP by disrupting the vicious cycle of lipid peroxidation and HO-1 activation, eventually ameliorating trabecular deterioration. This study provides a series of new targets and novel mechanisms for the treatment of DOP. Future studies should focus on the potential clinical applications of this therapeutic strategy.

## Materials and methods

### Chemical reagents and materials

PA and CTL BSA were purchased from Kunchuang Biotechnology (Xian, China). ZnPP (HY-101193) was purchased from MedChemExpress (Shanghai, China). STZ (S1312), Z-VAD-FMK (S7023), VitE (S4686), Nec-1 (S8037), 3-MA (S2767), DFO (S5742), and Fer-1 (S7243) were purchased from Selleck Chemicals (Shanghai, China). α-MEM cell culture medium and fetal bovine serum (FBS) were purchased from Gibco (USA).

### Animal experiments

All animal experiments were approved by the Animal Ethics Committee of Shanghai Ninth People’s Hospital (SH9H-2020-A249-1). Four-week-old male C57BL/6J mice were purchased from Shanghai Laboratory Animal Research Center (Shanghai, China) and housed under pathogen-free conditions at 24 ± 2 °C with 55% ± 5% humidity. A mouse model of type 2 diabetes was established according to a previously published study.^44^ After 1 week of adaptive feeding, mice were randomly divided into different groups for further analysis. To explore the skeletal phenotype of DOP mice, as shown in Fig. 1, mice were randomly divided into 2 groups: a normal control group (CTL; *n* = 6) and a type 2 DM model group (STZ&HFD; *n* = 6). To confirm the rescue effect of ZnPP and Fer-1 on DOP in mice, as shown in Fig. 7, mice were randomly divided into 4 groups: a normal control group (CTL; *n* = 6), a type 2 DM model group (STZ&HFD; *n* = 6), a ZnPP treatment group (STZ&HFD + ZnPP; *n* = 6) and a Fer-1 treatment group (STZ&HFD + Fer-1; *n* = 6). The animal experiment shown in Fig. 1 and the rescue experiment shown in Fig. 7 were performed independently using different individual animals. Mice in the CTL group were fed a standard diet (Research Diet, D12450B, 10% kcal from fat), and mice in the STZ&HFD, STZ&HFD + ZnPP, and STZ&HFD + ZnPP groups were fed a HFD (Research Diet, D12492, 60% kcal from fat) during the experiment. After feeding for 4 weeks, the mice in the STZ&HFD, STZ&HFD + ZnPP, and STZ&HFD + Fer-1 groups were fasted overnight and then injected i.p. with STZ (25 mg·kg^**−**1^ dissolved in 0.1 mmol·L^**−**1^ ice-cold citrate buffer, pH 4.6) for 4 days to induce type 2 DM. The mice in the CTL group were injected with citrate buffer as a negative CTL. One week after STZ injection, the mice in the STZ&HFD + ZnPP and STZ&HFD + Fer-1 groups were injected i.p. with ZnPP (10 mg·kg^**−**1^ dissolved in DMSO) or Fer-1 (1 mg·kg^**−**1^ dissolved in DMSO), respectively, twice weekly for 6 weeks. Additionally, the mice in the CTL and STZ&HFD groups were injected with DMSO. All mice were sacrificed for further analysis 8 weeks after STZ injection. For each mouse, body weight and fasting glucose were measured weekly until the end of the animal experiment.

### X-ray and micro-CT analyses

Plain radiography of the lower extremities of mice was carried out using a Faxitron MX20 X-ray system (Faxitron Bioptics, Tucson, USA). High-resolution micro-CT scanning of the lower extremities was performed using a Scanco μCT80 system (Scanco Medical, Brüttisellen, Switzerland).

### Histological analysis

IHC staining was performed as described previously.^45^ Briefly, mouse specimens were fixed with 4% buffered formalin for 48 h, decalcified for 28 days and embedded in paraffin. Embedded specimens were sectioned (5 μm thick) and stained with H&E. IHC staining was carried out with antibodies against GPX4 (Santa Cruz Biotechnology, sc-166570; dilution 1:100) and HO-1 (Proteintech, 10701-1-AP; dilution 1:200).

### TUNEL assay

Osteocyte death in bone tissue was assessed by TUNEL assays in situ using One-step TUNEL Assay Kits (C1086, Beyotime, Shanghai, China) strictly according to the instructions. Briefly, after deparaffinization and hydration, antigen repair was performed using proteinase K (20 μg·mL^**−**1^) at 37 °C for 15 min. Then, the tissue sections were incubated with TUNEL working solution at 37 °C for 60 min. Finally, the sections were stained with DAPI solution for 4 min at room temperature. Images were acquired using a confocal microscope (TCS SP8, Leica).

### Serum OCN, FFA, and PA measurement

The serum OCN concentration was assessed using a mouse OCN ELISA Kit (Qiyi Biotechnology, Shanghai, China), and the protocol was performed according to the manufacturer’s instructions. The serum FFA concentration was assessed using an FFA quantification kit from Solarbio (Beijing, China), and the serum PA concentration was assessed by high-performance liquid chromatography.

### Cell culture

The IDG-SW3 cell line was kindly provided by Dr. Lynda F. Bonewald. Cells grown in α-MEM supplemented with 10% FBS, 100 U·mL^**−**1^ penicillin, 100 μg·mL^**−**1^ streptomycin and 50 U·mL^**−**1^ IFN-γ at 33 °C in 5% CO_2_. During mineralization, IDG-SW3 cells were shifted to an environment of 37 °C in 5% CO_2_, and the culture medium was supplemented with 50 μg·mL^**−**1^ ascorbic acid and 4 mmol·L^**−**1^ β-glycerophosphate.

### CCK-8 assay

A CCK-8 (Bimake, Houston, USA) assay kit was used strictly according to the instructions to evaluate cell viability. Briefly, mineralized osteocytes were treated as indicated for 24 h, and the culture medium was then replaced with CCK-8 working solution containing 10% CCK-8 reagent. The cells were cultured at 37 °C for 1 h. The absorbance (450 nm) of each well was measured by a microplate reader.

### C11-BODIPY and FerroOrange staining

To prevent interference from Dmp1-GFP fluorescence in IDG-SW3 cells after mineralization induction, we used unmineralized IDG-SW3 cells for fluorescence staining. A C11-BODIPY probe (D3861, Invitrogen, USA) was used to evaluate cellular lipid peroxidation. After the indicated treatments, osteocytes were incubated with C11-BODIPY working solution (2.5 μmol·L^**−**1^) for 30 min prior to analysis. A FerroOrange probe (F374, Dojindo, Shanghai, China) was used to detect intracellular Fe^2+^. After the indicated treatments, osteocytes were washed with PBS solution and treated with FerroOrange working solution (1 μmol·L^**−**1^) for 30 min. Finally, stained cells were observed using confocal scanning microscopy.

### TEM

TEM was performed to observe alterations in mitochondrial morphology. After the indicated treatment, osteocytes were fixed with glutaraldehyde solution (2.5%, Electron Microscope Grade) at 4 °C for 16 h. After fixation, the cells were dehydrated, embedded, sectioned, and stained. Finally, the TEM samples were visualized using a Hitachi Model H-7650 transmission electron microscope.

### Iron measurement

Intracellular ferrous iron, ferric iron and total iron were assessed using an iron assay kit (MAK025, Sigma–Aldrich, USA). After the indicated treatments, mineralized osteocytes were harvested and lysed in iron assay buffer. The whole-cell lysate was centrifuged at 12 000 × *g* for 5 min, and only the supernatant was used in the iron measurement assay. The experimental procedure strictly followed the manufacturers’ instructions.

### MDA measurement

Serum and tissue MDA concentrations were assessed using a Lipid Peroxidation MDA Assay Kit (S0131, Beyotime, Shanghai, China). Intracellular MDA concentrations were assessed using a Cellular Lipid Peroxidation MDA Assay Kit (A003-4-1, Nanjing Jiancheng Bioengineering Institute, Jiangsu, China). After the indicated treatments, mineralized osteocytes were harvested and lysed in RIPA lysis solution. The cell lysate was centrifuged at 12 000 × *g* for 5 min, and the supernatant was collected for subsequent experiments. The MDA measurement procedure strictly followed the manufacturers’ instructions.

### RNA sequencing

Whole-transcriptome sequencing was performed using mineralized IDG-SW3 osteocytes treated with HGHF (glucose 25.5 mmol·L^**−**1^ + PA 300 μmol·L^**−**1^) or CTL BSA (glucose 5.5 mmol·L^**−**1^ + BSA 300 μmol·L^**−**1^) for 24 h. Three independent replicates per group were established. Total RNA was extracted by the standard TRIzol extraction method, and cDNA samples were sequenced using an Illumina NovaSeq 6000 system by Sinotech Genomics Co., Ltd (Shanghai, China). KEGG enrichment analysis, GSEA, and heatmap analysis were performed for all differentially expressed genes. *P* < 0.05 was considered to indicate a significant difference in KEGG enrichment analysis.

### RT-qPCR assay

RT-qPCR was conducted as previously described.^46^ Briefly, total RNA from each sample was extracted using TRIzol reagent and reverse transcribed with a Script™ Reverse Transcription Kit (A276A, Promega). SYBR Green qPCR Master Mix (B21202, Bimake) was used for RT–qPCR with a 7500 real-time PCR instrument (Thermo Fisher Scientific). The 2^−ΔΔCt^ method was used to calculate fold changes in mRNA expression. All primer sequences used are listed in Table S1.

### Cell transfection

For gene overexpression, the HO-1 overexpression plasmid (pcDNA3.1-HO-1-GFP), NRF2 overexpression plasmid (pcDNA3.1-NRF2-HA) and c-JUN overexpression plasmid (pcDNA3.1-c-JUN-Myc) were constructed by cloning the corresponding coding sequences into the pcDNA3.1(+) vector. The construction of the plasmids expressing the truncation mutants was performed according to a previous study.^47^ Lipofectamine^®^ 3000 reagent (Invitrogen, USA) was used for cell transfection according to the manufacturer’s instructions. Seventy-two hours after transfection, cells were harvested for further experiments.

For NRF2 and c-JUN knockdown, unmineralized osteocytes were transfected with siRNA against NRF2 or c-JUN using Lipofectamine^®^ 3000 reagent. Three different siRNA oligonucleotides were synthesized by Tsingke Biotechnology (Beijing, China) to target each gene. All siRNA oligonucleotide sequences used are listed in Table S2. Osteocytes were harvested for subsequent experiments 48 h after transfection.

### IF staining

To prevent interference from Dmp1-GFP fluorescence in IDG-SW3 cells after mineralization induction, we used unmineralized IDG-SW3 cells for IF staining. Osteocytes were fixed with cold 4% paraformaldehyde for 15 min, treated with 0.2% Triton for 5 min, blocked with 10% goat serum at room temperature for 60 min, and finally incubated with primary antibodies against c-JUN (Santa Cruz Biotechnology, sc-74543; mouse mAb, dilution 1:50) and NRF2 (CST, 12721; rabbit mAb, dilution 1:200) at 4 °C for 16 h. The next day, the samples were washed 3 times with TBST and incubated with anti-mouse IgG (CST; Alexa Fluor 594, dilution 1:1 000) and anti-rabbit IgG (CST; Alexa Fluor 488, dilution 1:1 000) secondary antibodies at room temperature for 60 min. Then, nuclei were stained with DAPI solution for 10 min. Confocal laser scanning microscopy was used for image acquisition.

### WB analysis and Co-IP

Total cellular protein was extracted using SDS lysis buffer (Beyotime, Shanghai, China) supplemented with protease and phosphatase inhibitor cocktail (Boster, Shanghai, China). Then, proteins were separated via electrophoresis, transferred to a 0.22-μm PVDF membrane, blocked using 5% BSA for 1 h and incubated with primary antibodies at 4 °C for 16 h. eBlot™ L1 Protein Transfer System (GenScript Corporation, China) was used for protein electrophoresis. The next day, the samples were washed 5 times with TBST and incubated with fluorescent secondary antibodies at room temperature for 1 h. Finally, proteins were detected using an Odyssey imaging system (Lincoln, NE).

Co-IP was performed with Protein A/G PLUS-Agarose (sc-2003, Santa Cruz, USA). Briefly, total cell lysates were extracted using NP-40 lysis buffer and separately incubated with primary antibodies or normal IgG at 4 °C for 16 h with gentle shaking. The next day, antibody–antigen complexes were captured with Protein A/G PLUS-Agarose. After several washes, the samples were boiled and analyzed by western blotting.

### In silico molecular modeling and docking of c-JUN to NRF2

An in silico docking study of c-JUN to NRF2 was performed as previously described.^48^ In brief, the crystal structure of c-JUN was aligned to the crystal structure of NRF2 using PyMOL software (http://www.pymol.org). Then, the docking study was performed using RosettaDock software (Rosetta Suite 3.4; http://rosie.graylab.jhu.edu/) for biased docking. Next, in the docking process, the initial prediction of the c-JUN:NRF2 protein complex was subjected to a rigid body docking search with flexible side chain optimization. One thousand independent simulations were performed, and all resulting protein complex models were ranked by interaction energy. Finally, the docking results were visualized using PyMOL software.

### ChIP assay

A SimpleChIP^®^ Plus Enzymatic Chromatin IP Kit (Magnetic Beads) (CST, USA) was used in the ChIP assay. Briefly, chromatin was crosslinked with 1% formaldehyde, digested into ~400 bp fragments, immunoprecipitated for 24 h with an anti-NRF2 antibody or normal IgG and finally pulled down using ChIP-grade magnetic beads. DNA spin columns from the ChIP assay kit were used for DNA purification. Enrichment of specific DNA sequences was assessed using RT-qPCR. The primers used in the ChIP-RT–qPCR assay are listed in Table S3.

### Luciferase reporter assay

The reporter plasmids Hmox1-WT-Luc and Hmox1-ΔAREs-Luc were constructed using the pGL4.10 vector (Promega). The luciferase reporter assay was performed as previously described.^49^ Briefly, osteocytes in 96-well plates were transfected with CTL vectors and reporter plasmids using Lipofectamine^®^ 3000 reagent. Various treatments were applied 24 h after transfection. Forty-eight hours after transfection, a dual-luciferase reporter assay kit (Beyotime, Shanghai, China) was used to detect luminescence signals according to the manufacturer’s instructions.

### Statistical analysis

All data are presented as the mean ± SD values. GraphPad Prism 8 software (San Diego, CA, USA) was used for statistical analysis. Student’s *t* test was used for comparisons between two groups. One-way or two-way ANOVA with Sidak’s multiple comparison test was used for comparisons among more than two groups. *P* < 0.05 was considered significant.

## Supplementary information

Supporting information
