# Supplementary material for: Targeting ferroptosis suppresses osteocyte glucolipotoxicity and alleviates diabetic osteoporosis
Source: Bone Res. 2022 Mar 9;10:26. doi: 10.1038/s41413-022-00198-w (PMC8904790; doi:10.1038/s41413-022-00198-w)
Supplement: Supplementary file 1 — Supporting information [file 41413_2022_198_MOESM1_ESM.docx]

Supporting Information

**Targeting Ferroptosis Alleviates Diabetic Osteoporosis via Suppressing Osteocyte Lipotoxicity**

*Yiqi Yang^1‡^, Yixuan Lin^1‡^, Minqi Wang^3^, Kai Yuan^1^, Qishan Wang^1^, Pei Mu^5^, Jingke Du^1^, Zhifeng Yu^1^, Shengbing Yang^1^, Kai Huang^1^, Yugang Wang^4^, Hanjun Li^2, 1*^ and Tingting Tang^1*^*

1 Shanghai Key Laboratory of Orthopaedic Implants, Department of Orthopaedic Surgery, Shanghai Ninth People’s Hospital, Shanghai Jiao Tong University School of Medicine, Shanghai

2 Clinical Stem Cell Research Center, Renji Hospital, Shanghai Jiao Tong University School of Medicine, Shanghai

3 Department of Bone and Joint Surgery, Renji Hospital, School of Medicine, Shanghai Jiao Tong University, Shanghai

4 Department of Trauma Surgery, Department of Orthopedics, Renji Hospital, School of Medicine, Shanghai Jiao Tong University, Shanghai

5 Department of Orthopaedics, Shanghai Jiangong Hospital, Shanghai

‡These authors contributed equally: Yiqi Yang and Kai Yuan

Corresponding Authors:

*E-mail (Tingting Tang): [ttt@sjtu.edu.cn](mailto:ttt@sjtu.edu.cn)

*E-mail (Hanjun Li): [hanklee@sjtu.edu.cn](mailto:hanklee@sjtu.edu.cn)

**Fig. S1** Verification of the DOP mouse model

**Fig. S2** Cortical analysis of tibia using micro-CT

**Fig. S3** Quantitative analysis of GPX4 and PTGS2 expression *in vivo*

**Fig. S4** The effect of Fer-1 on osteocyte viability at different concentrations

**Fig. S5** DFO decelerated HGHF-induced osteocyte ferroptosis

**Fig. S6** Volcano plot of RNA-sequencing

**Fig. S7** Validation of HO-1 overexpression

**Fig. S8** Quantitative analysis of c-JUN and NRF2 expression *in vivo*

**Fig. S9** The interaction between NRF2 and c-JUN in the absence of HGHF

**Table S1** Primer sequences used in RT-qPCR

**Table S2** Sequences of siRNA used in cell transfection

**Table S3** Primer sequences used in ChIP-RT-qPCR


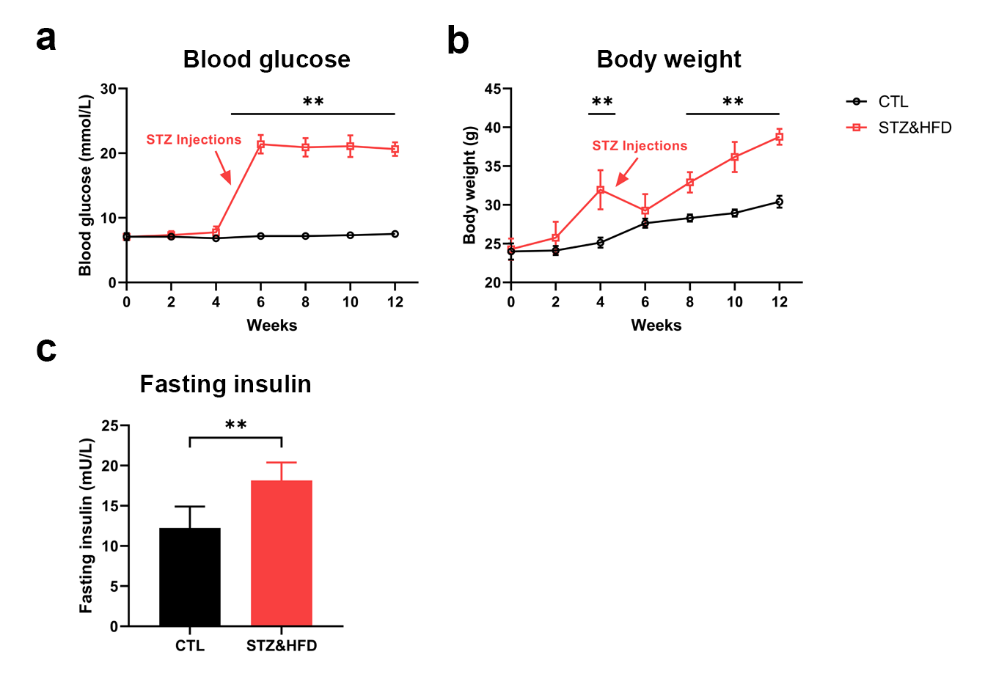


**Fig. S1** Verification of the DOP mouse model. The DOP model was generated by an HFD with low doses of STZ injection. Blood glucose (**a**) and body weight (**b**) were assessed biweekly. **c** Fasting insulin concentration was determined on the same day of euthanasia. “**” indicates p < 0.01. Each group contained n = 6 mice.


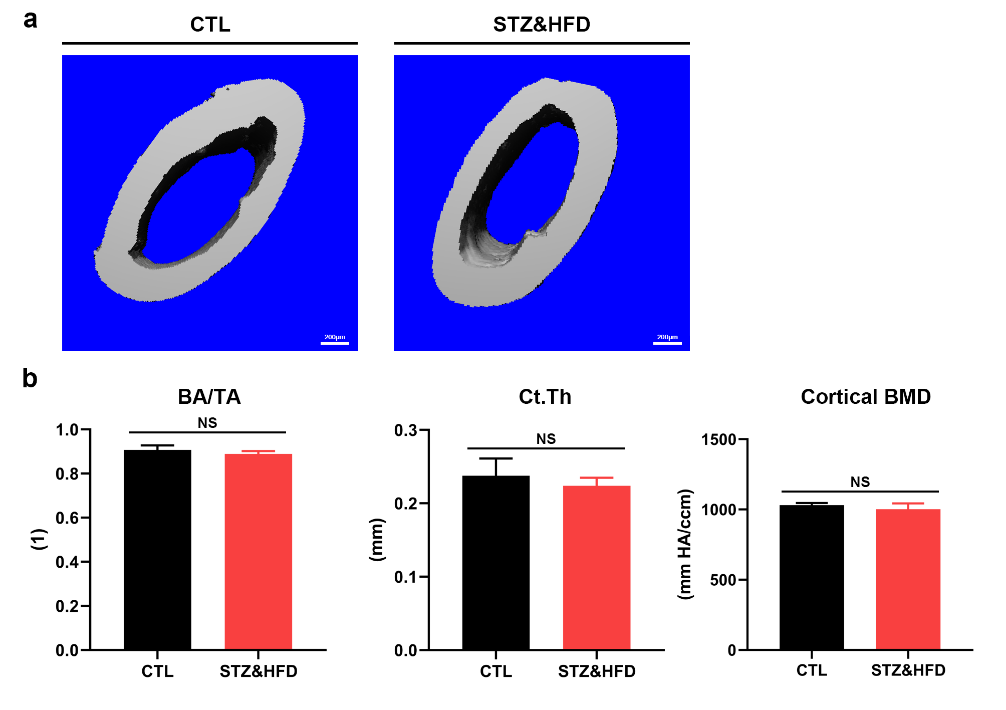


**Fig. S2** Cortical analysis of tibia using micro-CT. **a** Representative micro-CT radiographs of the femoral midshaft. **b** Quantitative analysis of cortical bone parameters. “NS” indicates not significant. Each group contained n = 6 mice.


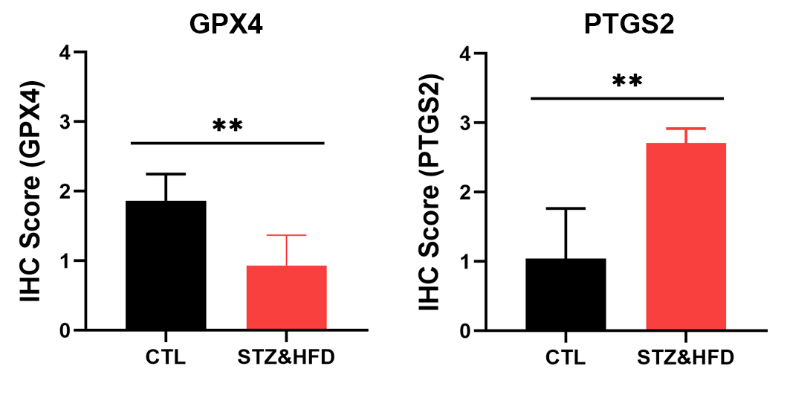


**Fig. S3** Quantitative analysis of GPX4 and PTGS2 expression *in vivo*. Each group contained at least n = 3 mice for quantitative analysis.


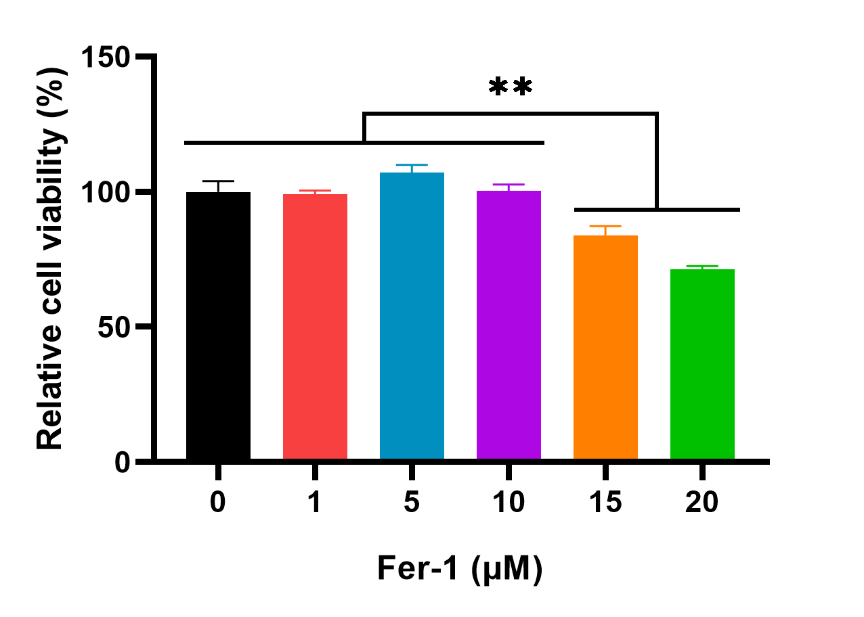


**Fig. S4** The effect of Fer-1 on osteocyte viability at different concentrations.


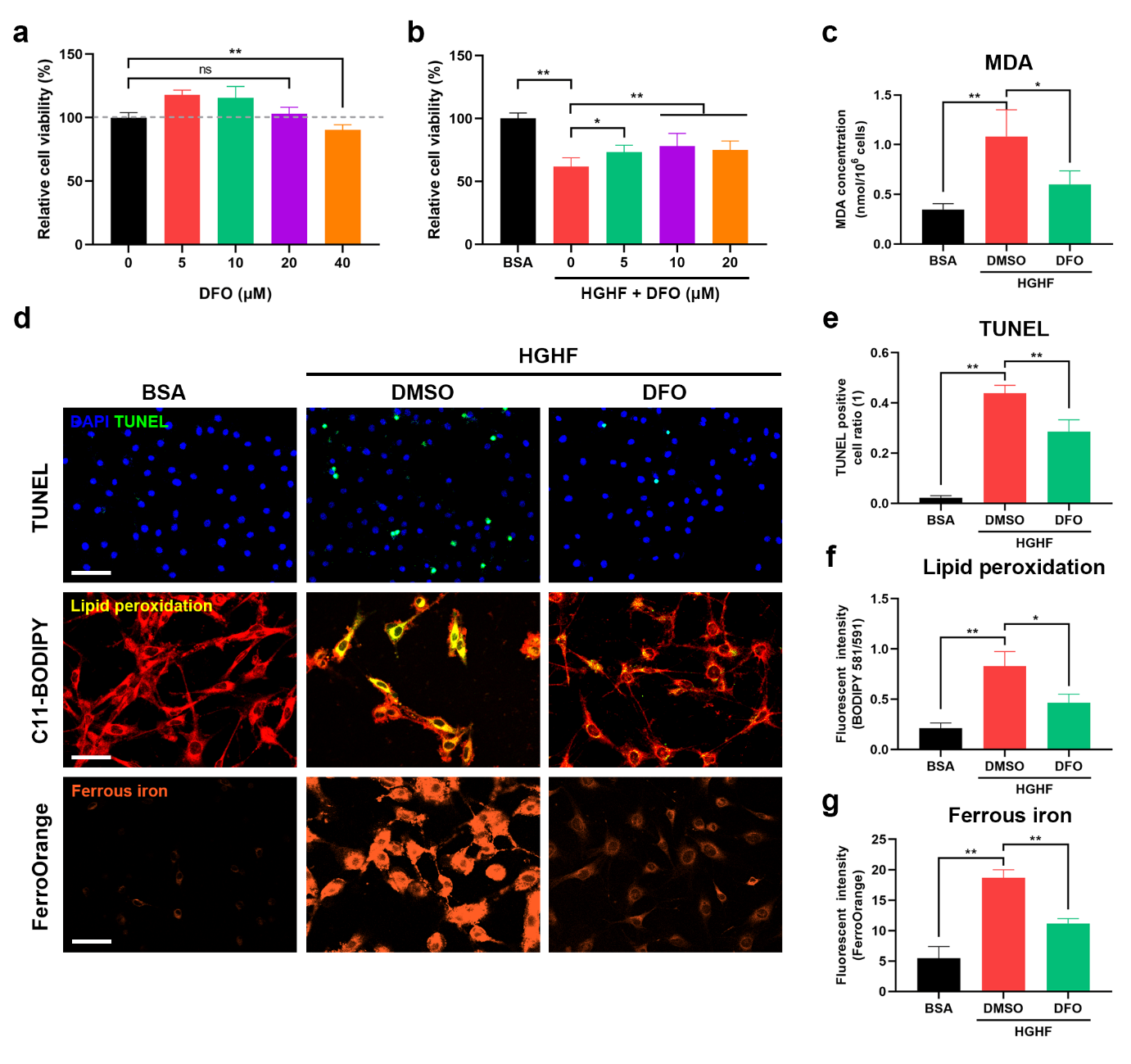


**Fig. S5** DFO decelerated HGHF-induced osteocyte ferroptosis. **a** CCK-8 assay of osteocytes treated with various concentrations of DFO for 24 h. **b** The rescue effect of DFO within the cell-friendly concentration range on osteocytes under HGHF treatment. **c** The level of MDA in osteocytes was quantitatively determined using an MDA assay kit. **d** Osteocytes were treated with BSA, HGHF or HGHF + DFO for 24 h. Representative images of TUNEL staining (scale bar: 100 μm), C11-BODIPY staining (scale bar: 100 μm) an FerroOrange staining (scale bar: 100 μm) are presented. **e** Semiquantitative analysis of TUNEL-positive cells. Semiquantitative analysis of the fluorescence intensity of lipid peroxidation (**f**) and ferrous iron (**g**). “*” indicates p < 0.05; “**” indicates p < 0.01. All data were from n = 3 independent experiments.


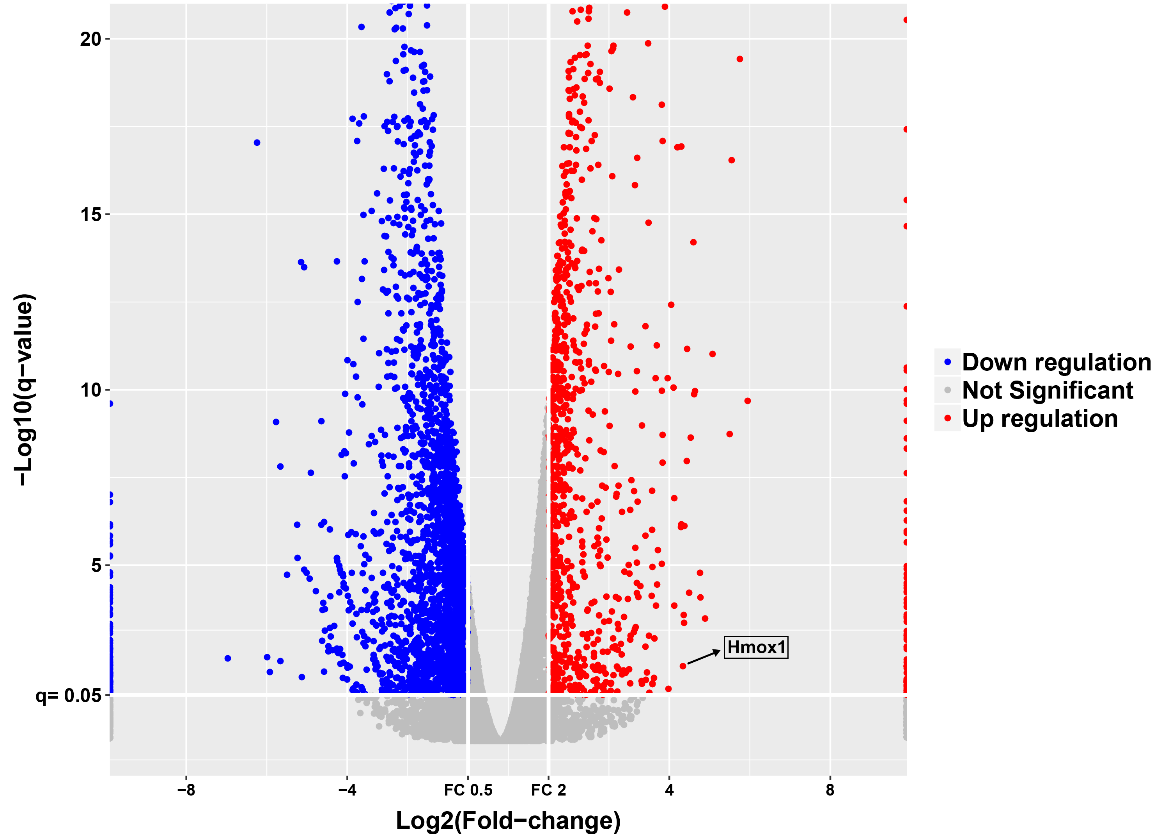


**Fig. S6** Volcano plot of RNA sequencing. Volcano plot illustrating the genes with upregulated (red dots) and downregulated (blue dots) expression induced by HGHF treatment based on the RNA sequencing analysis. The black arrow indicates the Hmox1 gene.


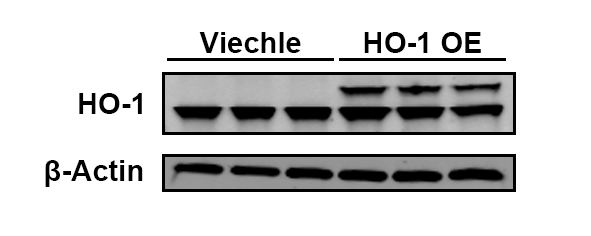


**Fig. S7** Validation of HO-1 overexpression. The efficiency of HO-1 overexpression was verified using western blotting.


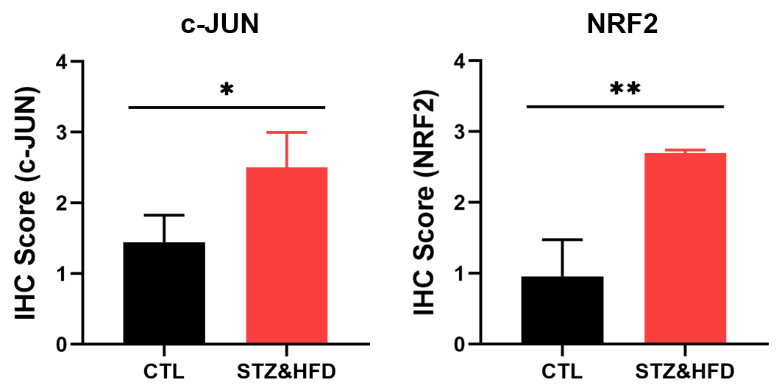


**Fig. S8** Quantitative analysis of c-JUN and NRF2 expression *in vivo*. Each group contained at least n = 3 mice for quantitative analysis.


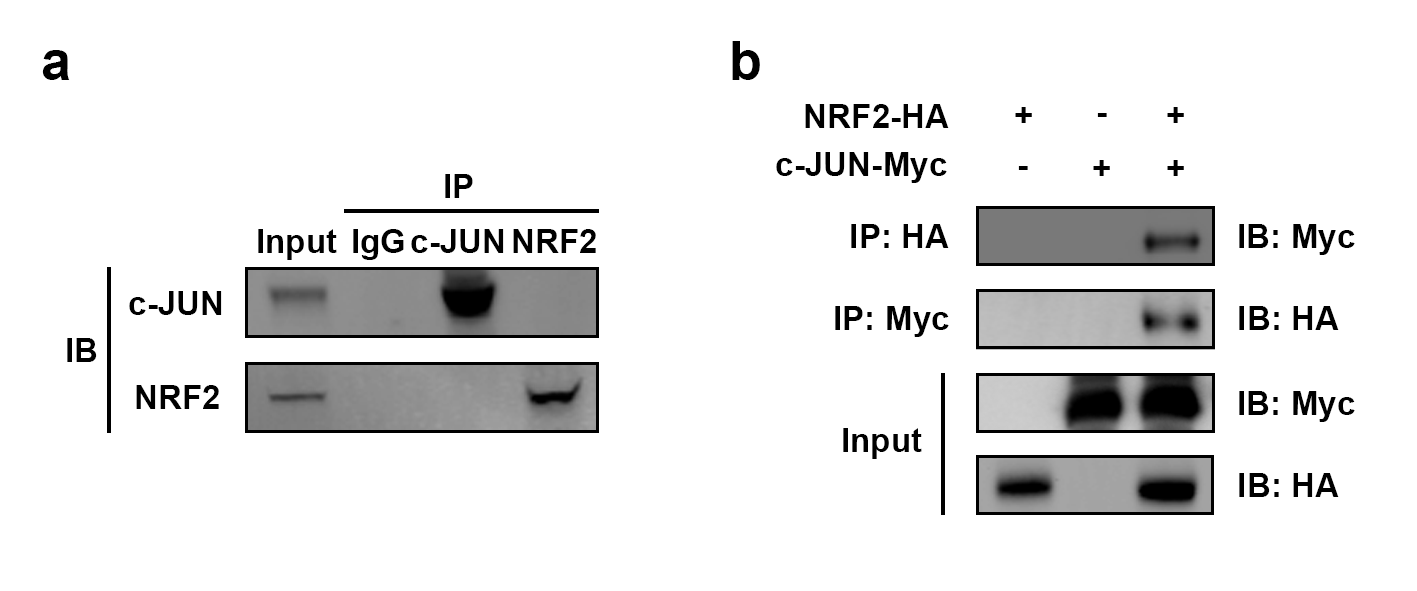


**Fig. S9** The interaction between NRF2 and c-JUN in the absence of HGHF. **a** Co-IP results of endogenous NRF2 and c-JUN in osteocytes without HGHF treatment. **b** Co-IP results of NRF2 and c-JUN in the osteocytes transfected with the NRF2-HA and c-JUN-Myc plasmids without HGHF treatment. All data were from n = 3 independent experiments.

**Table S1.** Primer sequences used in RT-qPCR

| Gene | Sequence (5’ - 3’) | |
| --- | --- | --- |
| Tfrc | Forward | GTTTCTGCCAGCCCCTTATTAT |
|  | Reverse | GCAAGGAAAGGATATGCAGCA |
| Slc11a2 | Forward | CAATGTCTTTGTCGTGTCCGT |
|  | Reverse | GCGACCATTTTAGGTTCAGGAAT |
| Sat1 | Forward | GAGAACACCCCTTCTACCACT |
|  | Reverse | GCCTCTGTAATCACTCATCACGA |
| Ptgs2 | Forward | GACAGATCATAAGCGAGGA |
|  | Reverse | CTCCACCAATGACCTGATAT |
| Nox1 | Forward | GGTTGGGGCTGAACATTTTTC |
|  | Reverse | TCGACACACAGGAATCAGGAT |
| Hmox1 | Forward | AAGCCGAGAATGCTGAGTTCA |
|  | Reverse | GCCGTGTAGATATGGTACAAGGA |
| Gpx4 | Forward | CTGGGAAATGCCATCAAAT |
|  | Reverse | GTCCTTCTCTATCACCTGG |
| Fth1 | Forward | CAAGTGCGCCAGAACTACCA |
|  | Reverse | GCCACATCATCTCGGTCAAAA |
| Acsl4 | Forward | AGCGTTCCTCCAAGTAGA |
|  | Reverse | GCCTGTCATTCCAGCAAT |

**Table S2.** Sequences of siRNA used in cell transfection

| Gene | Sequence (5’ - 3’) | |
| --- | --- | --- |
| c-jun-si-1 | Forward | GGCACAGCUUAAGCAGAAA |
|  | Reverse | UUUCUFCUUAAGCUGUGCC |
| c-jun-si-2 | Forward | GGUGCCUACGGCUACAGUA |
|  | Reverse | UACUGUAGCCGUAGGCACC |
| c-jun-si-3 | Forward | GGAUCAAGGCAGAGAGGAA |
|  | Reverse | UUCCUCUCUGCCUUGAUCC |
| Nrf2-si-1 | Forward | CCGAAUUACAGUGUCUUAA |
|  | Reverse | UUAAGACACUGUAAUUCGG |
| Nrf2-si-2 | Forward | CUCGCAUUGAUCCGAGAUA |
|  | Reverse | UAUCUCGGAUCAAUGCGAG |
| Nrf2-si-3 | Forward | CAAGGAGCAAUUCAAUGAA |
|  | Reverse | UUCAUUGAAUUGCUCCUUG |

**Table S3.** Primer sequences used in ChIP-RT-qPCR

| Region | Forward primer (5’-3’) | Reverse primer (3’-5’) |
| --- | --- | --- |
| A (AREs) | CCACAGGAGCTGAACTTTGT | TGAGGCTGAGGGAACAG |
| B (Exon 3) | TGATGGCTTCCTTGTACCATATC | AGCTCCTCAGGGAAGTAGAG |
